# Cecal cancer with essential thrombocythemia treated by laparoscopic ileocecal resection: a case report

**DOI:** 10.1186/s40792-019-0660-3

**Published:** 2019-06-21

**Authors:** Masaya Hiyoshi, Hiroaki Nozawa, Kentaro Inada, Takayoshi Koseki, Keiichi Nasu, Yasuji Seyama, Ikuo Wada, Koji Murono, Shigenobu Emoto, Manabu Kaneko, Kazuhito Sasaki, Yasutaka Shuno, Takeshi Nishikawa, Toshiaki Tanaka, Keisuke Hata, Kazushige Kawai, Tsuyoshi Maeshiro, Sachio Miyamoto, Soichiro Ishihara

**Affiliations:** 1 Department of Surgical Oncology, Faculty of Medical Sciences, the University of Tokyo, 7-3-1 Hongo, Bunkyo-ku, Tokyo, 113-8655 Japan; 2 Department of Surgery, Tokyo Metropolitan Bokutoh Hospital, Tokyo, Japan

**Keywords:** Anagrelide, Colorectal cancer, Essential thrombocythemia, Laparoscopic surgery

## Abstract

**Background:**

Essential thrombocythemia (ET) is a myeloproliferative disorder characterized by thrombocytosis and a propensity for both thrombotic and hemorrhagic events. ET rarely occurs simultaneously with colorectal cancer. Here, we report a case of colorectal cancer in an ET patient treated using laparoscopic ileocecal resection.

**Case presentation:**

A 40-year-old woman was admitted to our hospital after presenting with liver dysfunction. She had been previously diagnosed with ET; aspirin and anagrelide had been prescribed. Subsequent examination at our hospital revealed cecal cancer. Distant metastasis was absent; laparoscopic ileocecal resection was performed. Anagrelide was discontinued only on the surgery day. She was discharged on the seventh postoperative day without thrombosis or hemorrhage. However, when capecitabine and oxaliplatin were administered as adjuvant chemotherapy with continued anagrelide administration, she experienced hepatic dysfunction and thrombocytopenia; thus, anagrelide was discontinued. Five days later, her platelet count recovered. Subsequently, anagrelide and aspirin administration was resumed, without any adjuvant chemotherapy. Her liver function normalized gradually in 4 months. One-year post operation, she is well without tumor recurrence or new metastasis.

**Conclusions:**

To our knowledge, this is the first report of laparoscopic colectomy performed on an ET patient receiving anagrelide. Our report shows that complications such as bleeding or thrombosis can be avoided by anagrelide administration. Contrastingly, thrombocytopenia due to anagrelide intake should be considered when chemotherapy that could cause bone marrow suppression is administered.

## Background

Essential thrombocythemia (ET) is a myeloproliferative disorder characterized by thrombocytosis and a propensity for both thrombotic and hemorrhagic events [1]. ET treatment aims to prevent thrombotic and hemorrhagic complications and alleviate symptoms [2]. Low-dose aspirin is administered to reduce arterial thrombotic event risk [1]. Hydroxyurea and anagrelide are used for controlling platelet counts [2].

Colorectal cancer concurrent with ET is extremely rare. Here, we report a case of colorectal cancer in an ET patient treated using laparoscopic ileocecal resection.

## Case Presentation

A 40-year-old female presenting with mild hepatic dysfunction was referred to our hospital. She did not smoke but had a drinking habit. At age 28 years, she had presented with elevated platelet counts (> 100 × 10^4^/μL); ET had been diagnosed based on bone marrow biopsy results. She was prescribed aspirin (100 mg/day) and anagrelide (2.5 mg/day). She had also been prescribed ebastine for itching a while ago.

**Fig. 1:**
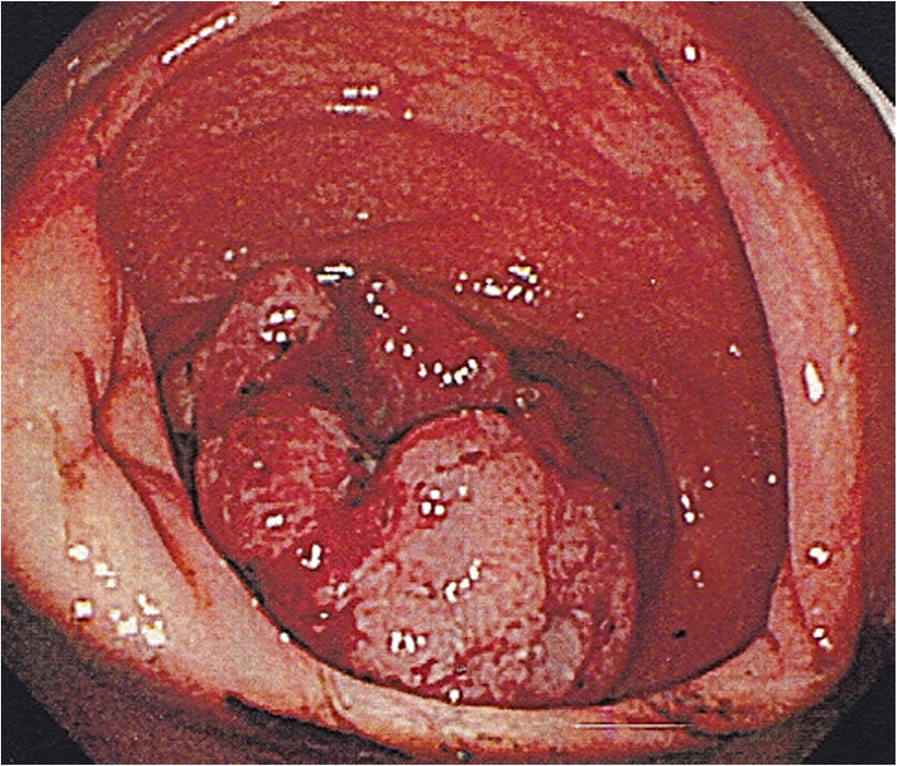
Colonoscopy showing a type 2 tumor in the cecum

**Fig. 2:**
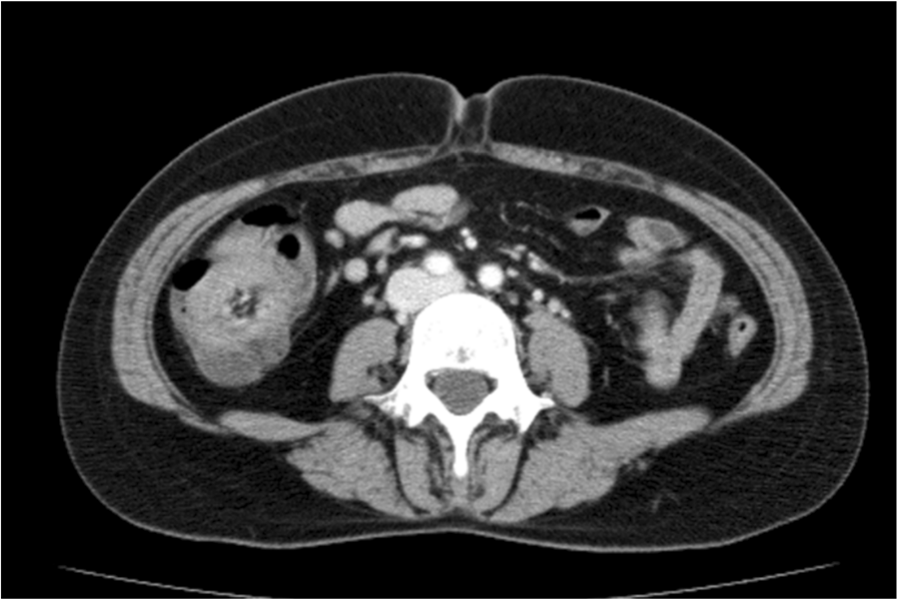
Abdominal computed tomography showing cecal wall thickening

On admission to our hospital, laboratory examination revealed slightly elevated alanine aminotransferase (ALT) levels (82 IU/L), although the patient’s ALT level had improved from that recorded previously. Her platelet count was slightly elevated (62.4 × 10^4^/μL). Prothrombin time and activated partial thromboplastin time were normal. Abdominal ultrasonography revealed a cecal tumor. Colonoscopy revealed advanced cecal cancer (Fig. 1). Computed tomography (CT) indicated cecal wall thickening (Fig. 2).

The patient recovered from liver dysfunction without treatment. She stopped taking oral aspirin 1 week prior to surgery but continued anagrelide until the day before surgery. To prevent thrombosis, she wore elastic stockings; furthermore, intermittent pneumatic compression was performed during surgery. Laparoscopic-assisted ileocecal resection was performed. We used a soft coagulation system to achieve complete hemostasis. The operative duration was 202 min; blood loss was 34 mL.

**Fig. 3:**
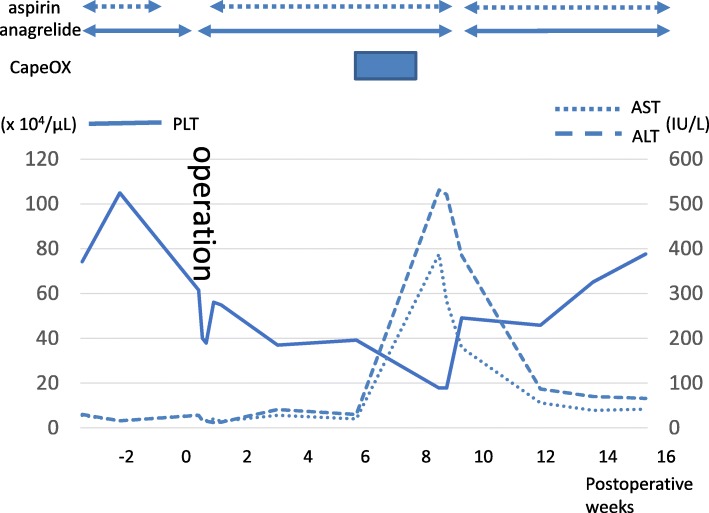
Transition of platelet counts and transaminase levels from surgery to end of adjuvant chemotherapy. Anagrelide was discontinued only on the operation day and restarted on the first postoperative day. After one course of intravenous oxaliplatin plus oral capecitabine, platelets decreased and hepatic transaminase level increased. Thus, aspirin and anagrelide were discontinued; platelet count recovered to 50 × 10^4^/μL after 5 days. She resumed internal use of anagrelide and aspirin but did not restart adjuvant chemotherapy; liver transaminase levels normalized gradually

From the first postoperative day, the patient started walking, drinking water, and resumed oral anagrelide intake. She resumed oral aspirin intake on the fifth postoperative day. Her perioperative platelet count was controlled to approximately 40–60 × 10^4^/μL (Fig. 3). Prothrombin time and activated partial thromboplastin time also did not show abnormal values during the perioperative period. The postoperative course was uneventful and she was discharged on the seventh postoperative day.

The tumor pathological stage was T3N1M0 (Stage IIIB). The patient received intravenous oxaliplatin plus oral capecitabine (CapeOX) as postoperative adjuvant chemotherapy (oxaliplatin 130 mg/m^2^, capecitabine 1000 mg/m^2^). However, after one course, she again experienced liver dysfunction (aspartate aminotransferase [AST] level, 388 IU/L; ALT level, 531 IU/L); because of anagrelide, her platelet count decreased to 17.8 × 10^4^/μL. Therefore, we asked her to discontinue anagrelide and aspirin that day onwards; 5 days later, her platelet count recovered to 50 × 10^4^/μL. Subsequently, she resumed taking anagrelide and aspirin; however, she refused to resume any adjuvant chemotherapy after this incident. Her liver function normalized gradually in 4 months. There were no clinical signs of thrombosis, and there was no appearance of a new thrombus on contrast-enhanced CT 6 months after the operation. One-year post operation, she is well without tumor recurrence or new metastasis.

## Conclusions

ET is a myeloproliferative disorder characterized by excess platelet production [2]. ET patients have increased risk of postoperative bleeding and thrombosis [3]. Riggeri et al. reported a retrospective survey of postoperative outcomes of polycythemia vera and ET patients [4]. In their cohort, 23 (7.3%) of 311 patients who underwent surgical interventions experienced major hemorrhagic episodes [4]. This rate was higher than that observed in normal cancer surgery (about 1%) [5]. Zhu et al. reported a case involving an ET patient with sigmoid colon cancer. Plateletpheresis was performed before surgery but anastomotic bleeding complicated the postoperative course. A systematic review of abdominal operations in four ET patients revealed that two of four patients experienced bleeding complications on the first postoperative day [3].

In laparoscopic surgery, pneumoperitoneum, the Trendelenburg position, and long surgical time could be risk factors for lower extremity venous stasis [6]. However, a meta-analysis of trials comparing laparoscopic surgery with open surgery demonstrated that laparoscopic surgery could achieve the same outcome as open surgery with regards to presence of postoperative venous thromboembolism [6]. However, to prevent thrombosis, we performed laparoscopic surgery in the leg-open position, instead of lithotomy, using an elastic stocking and intermittent pneumatic compression with pneumoperitoneum pressure of up to 8 mmHg. Small amounts of bleeding have been reported in laparoscopic surgery due to pneumoperitoneum pressure; however, treatment for ET patients involves a high bleeding risk as described above, so we also performed careful hemostasis confirmation before closing. The patient’s wound was small and less painful; she resumed walking on the first postoperative day and recovered quickly after surgery. Postoperative administration of Xa inhibitors was not generally indicated at the time, as was she, but she was fortunately discharged without complications. However, the optimal perioperative management of ET patients remains unclear, especially in the context of abdominal malignancy.

A high platelet count (> 60 × 10^4^/μL), age > 60 years, and thrombosis history are high-risk factors for major thrombosis in ET patients [7]. Patients at high thrombosis risk should be treated with cytoreductive therapy (CRT) to reduce thrombosis risk. Presently, two CRT treatments are licensed in Japan: hydroxyurea and anagrelide [2]. Anagrelide is an orally active, platelet-lowering agent approved for first-line treatment of high-risk ET in Japan [2]. Hydroxyurea is most widely used for CRT, although long-term use may be associated with a high secondary leukemia incidence [2]. Recently, Kanakura et al. reported anagrelide efficacy in high-risk ET patients in Japan [2]. Anagrelide was approved for ET treatment in the US and Europe in 1997 and 2004, respectively, but it was not licensed in Japan until 2014. Our patient was prescribed anagrelide about 1 year before surgery; her platelet count was properly controlled. To our knowledge, ours is the first case involving laparoscopic colectomy in a patient who used anagrelide for platelet count management.

CapeOX as adjuvant therapy has a manageable tolerability profile and should be considered as a standard adjuvant treatment option in stage III colorectal cancer patients. Transient thrombocytopenia is relatively common in oxaliplatin-treated patients. Hepatotoxicity is another adverse event observed with oxaliplatin-based chemotherapy [8]. Here, we encountered a considerably decreased platelet count on starting chemotherapy with ongoing anagrelide intake. Hashiba et al. reported the outcome of discontinuing CRT in an ET patient during chemotherapy for esophageal cancer. They administered preoperative chemotherapy (cisplatin + 5-fluorouracil) accompanied by hydroxyurea discontinuation; however, platelet count increased to 116 × 10^4^/μL after therapy [9]. Since our patient was relatively young, we suggested a method of carefully re-administering fluoropyrimidine monotherapy as adjuvant chemotherapy. However, she did not wish to resume treatment because of concerns about adverse events. Thus, the tolerability of fluoropyrimidine monotherapy is unknown as well. Chemotherapeutic strategies for ET patients should consider various events such as bone marrow suppression due to chemotherapy, thrombocytopenia caused by CRT, and elevated platelet count due to drug withdrawal.

Thus, we reported on a patient with colon cancer and ET, which are rarely observed together, who required careful perioperative management for laparoscopic surgery. We found that complications such as bleeding or thrombosis during control of the platelet count can be avoided by anagrelide administration. Contrastingly, thrombocytopenia due to anagrelide intake should be considered when chemotherapy that could cause bone marrow suppression is administered. Further studies involving this unique group of patients should focus on developing appropriate treatment strategies.

## Data Availability

The authors declare that all the data related to this article are available in this manuscript.

## Abbreviations

ALT: Alanine aminotransferase
AST: Aspartate aminotransferase
CT: Computed tomography
CapeOX: Intravenous oxaliplatin plus oral capecitabine
CRT: Cytoreductive therapy
ET: Essential thrombocythemia
